# The Differential Phosphorylation-Dependent Signaling and Glucose Immunometabolic Responses Induced during Infection by *Salmonella* Enteritidis and *Salmonella* Heidelberg in Chicken Macrophage-like cells

**DOI:** 10.3390/microorganisms8071041

**Published:** 2020-07-14

**Authors:** Famatta Perry, Casey Johnson, Bridget Aylward, Ryan J. Arsenault

**Affiliations:** Department of Animal and Food Sciences, University of Delaware, Newark, DE 19716, USA; famper@udel.edu (F.P.); johnsocn@udel.edu (C.J.); baylward@udel.edu (B.A.)

**Keywords:** immunometabolism, *Salmonella* Enteritidis, *Salmonella* Heidelberg, chickens, macrophages

## Abstract

*Salmonella* is a burden to the poultry, health, and food safety industries, resulting in illnesses, food contamination, and recalls. *Salmonella enterica* subspecies *enterica* Enteritidis (*S.* Enteritidis) is one of the most prevalent serotypes isolated from poultry. *Salmonella enterica* subspecies *enterica* Heidelberg (*S.* Heidelberg), which is becoming as prevalent as *S.* Enteritidis, is one of the five most isolated serotypes. Although *S.* Enteritidis and *S.* Heidelberg are almost genetically identical, they both are capable of inducing different immune and metabolic responses in host cells to successfully establish an infection. Therefore, using the kinome peptide array, we demonstrated that *S.* Enteritidis and *S.* Heidelberg infections induced differential phosphorylation of peptides on Rho proteins, caspases, toll-like receptors, and other proteins involved in metabolic- and immune-related signaling of HD11 chicken macrophages. Metabolic flux assays measuring extracellular acidification rate (ECAR) and oxygen consumption rate (OCR) demonstrated that *S.* Enteritidis at 30 min postinfection (p.i.) increased glucose metabolism, while *S.* Heidelberg at 30 min p.i. decreased glucose metabolism. *S.* Enteritidis is more invasive than *S.* Heidelberg. These results show different immunometabolic responses of HD11 macrophages to *S.* Enteritidis and *S.* Heidelberg infections.

## 1. Introduction

*Salmonellae* are rod-shaped, Gram-negative, facultative anaerobes [1] and are the number one cause of foodborne gastroenteritis [2,3]. The species *Salmonella enterica* is a highly diverse bacterial species consisting of six subtypes and over 2500 serovars [1,4]. *Salmonella enterica* subspecies *enterica* Enteritidis (*S.* Enteritidis) and *Salmonella enterica* subspecies *enterica* Heidelberg (*S.* Heidelberg) are two of the many serovars under the subspecies that cause nontyphoidal salmonellosis (NTS) resulting in gastroenteritis [5,6,7,8]. *Salmonellae* that can be transmitted from animals, humans, and plants are referred to as nonspecific serovars (nonrestricted) [1,4,9]. Reported cases of NTS poisoning include acute gastroenteritis and watery diarrhea that occur approximately 6–12 h after ingestion or contraction of bacteria in humans [4,9]. Other common symptoms may include nausea, vomiting, abdominal pain, and fever [4]. NTS symptoms usually last 4 to 10 days and may resolve on their own with time [10]. NTS can also become invasive in humans, that is, *Salmonella* can enter cells, replicate, and spread quickly. Sometimes *Salmonella* enters the bloodstream and other organs of the host, causing severe illness [5]. This happens when the pathogen is contracted by an individual that is immunocompromised [11] or has a weak/undeveloped immune system (such as in the case of children and elderly people) [4,8,11].

*S.* Enteritidis and *S.* Heidelberg are among the serovars with the highest recovery rates and two of the three serovars commonly related to NTS [1,12]. Although nonpathogenic to chickens, *S.* Enteritidis was recognized as the most prevalent *Salmonella* isolate in poultry in the 1990s to mid-2000s [1] and is one of the major serovars in poultry now [10,13]. *S.* Enteritidis reservoirs are not limited to poultry. *S.* Enteritidis has been isolated from, pigs, cattle, and plants [9,14], which are major factors contributing to *Salmonella* outbreaks leading to illnesses, hospitalizations, and even death of humans [8,11,15].

Like *S.* Enteritidis, *S.* Heidelberg has been a major concern for many decades because it is infectious to humans [16]; within the past 10 years, there has been an increase in its prevalence in chicken [17]. Moreover, similar to *S.* Enteritidis and many food-related NTS serovars, *S.* Heidelberg has maintained its prevalence on farms and in poultry, beef, and pork due to carriers such as rodents and other farm pests [9,14].

An avian macrophage cell line has been used by poultry researchers to study immune responses to *Salmonella* infections and other pathogens [18,19,20]. The avian macrophage cell line known as HD11 chicken macrophage-like cells are avian myelocytomatosis type MC29 virus transformed chicken hematopoietic cells that display the surface antigen markers and phenotypic function of macrophages [20,21]. Similar to macrophages in vivo, HD11 cells have been shown to phagocytize bacteria [20,21,22]. For example, a study performed by Wisner et al. also showed that HD11 cells can phagocytize different strains of *Salmonella* [22].

Macrophages are an important component of the innate immune system and play a vital role in responding to bacterial invasion [23]. Macrophages play a central role in the innate immune defense of the host by recognizing and killing pathogens [23,24]. One reason that macrophages were chosen for this research is the recognition that macrophages serve as hosts to *Salmonella* [25]. *Salmonella* can survive and replicate in a cell whose role is to destroy bacteria. Macrophages are present in many distinct tissues of the host, including the gut [25,26]. Macrophages are known to exist in two main states, the inflammatory state (M1) and the anti-inflammatory state (M2) [27,28]. Researchers have shown that the M1 state is closely associated with the upregulation and activation of glycolytic proteins, reactive oxygen species (ROS), and inflammatory cytokines (tumor necrosis factor (TNF)-alpha, interleukin (IL)-12, IL-17, etc.), while the M2 cells undergo fatty acid oxidation, immune suppression (increasing levels of TGF-beta and IL-10), and cell repair [24,28]. Using the information available about macrophages, their control of pathogens, and their response to intracellular invasion, we aimed to expand our understanding of the metabolic changes over time that render these important immune cells susceptible to *Salmonella* infections. The metabolic status of immune cells during an immune response and the energetic processes that drive the production of cytokines play a crucial role in an organism’s overall immunity. This cross-talk between the immune and metabolic system is known as immunometabolism [29]. This paper focuses on the immune and metabolic changes that occur in the cell during *Salmonella* infections.

Our laboratory employs kinome peptide array analysis to determine changes in cellular processes. This technique measures phosphorylation, a post-translational modification of proteins [29,30]. Phosphorylation is carried out by enzymes known as kinases to induce changes in proteins that affect cellular function. The kinome peptide array allows the visualization of peptides phosphorylated by kinases in a biological sample and the determination of the changes induced in protein functions, cellular processes, and pathways [31,32]. Since many signaling processes within a cell are dependent on phosphorylation by kinases, recognizing specific kinase target sequences and the specific serine, threonine, or tyrosine residues within those sequences subject to phosphorylation allows us to determine the change in functionality of the protein and thus the change in cell response. Addition of phosphate groups by kinases can result in the activation or deactivation of proteins that control different signals in a cell [29,33,34].

Studies have shown that *S.* Enteritidis and *S.* Heidelberg induce different responses in chicken hosts in vivo [35] and in vitro [18], including significantly altering the phosphorylation of mammalian target of rapamycin (mTOR) and 5′ adenosine monophosphate-activated protein kinase (AMPK) [36]. The study described here focuses on the intracellular responses of chicken hosts to infection by these two different serovars of *Salmonella* at different timepoints in vitro. With the kinome peptide array technology, we defined the changes in phosphorylation of key immune and metabolic response proteins and how these changes affect cellular function. We validated the indicated changes in cellular responses using other molecular-based techniques. Understanding the immunometabolic changes that the bacteria induces on the host and subsequent host responses highlights key mechanisms of infection in the avian immune system. This understanding would serve as a basis for potential intervention strategies toward treatment of infections by various serovars of *Salmonella* in poultry.

## 2. Materials and Methods

### 2.1. Cell Line and Maintenance

HD11 cells are referred to as chicken macrophage-like cells because they represent an immortalized bone marrow derived cell line that is transformed with the avian myelocytomatosis type MC29 virus [18]. The cells were maintained in cell culture media containing Iscove’s Modified Dulbecco’s Media (IMDM) (GE Life Sciences, Logan, UT, USA) with 10% fetal bovine serum (Midsci, Valley Park, MO, USA) and 1% 1.5 mM L-glutamine (containing penicillin and streptomycin) (Gibco, Grand Island, NY, USA) at 37 °C, 5% CO_2_, and 95% humidity. When required, cells were counted using a hemocytometer and a dilution factor of 10 in trypan blue (Sigma-Aldrich, St. Louis, MO, USA). HD11 cells were obtained from the laboratory of Dr. Mark Parcells, University of Delaware.

### 2.2. Bacteria Serovars

Serotyped *S.* Enteritidis and serotyped *S.* Heidelberg from infected chickens were obtained from Dr. Haiqi He, US Department of Agriculture Research Service. Both *S.* Enteritidis and *S.* Heidelberg were designed to be resistant to nalidixic acid and novobiocin [37]. The *Salmonella* stocks were stored in tryptic soy broth (TSB (Becton, Dickinson and Company, Sparks, MD, USA)) and 20% glycerol (Sigma-Aldrich, St. Louis, MO, USA) at −80 °C. Stocks were thawed, and 100 µL was cultured in a shaker at 37 °C in an Erlenmeyer flask containing 30 mL of TSB with antibiotics (25 µg/mL novobiocin and 20 µg/mL nalidixic acid (Sigma-Aldrich, St. Louis, MO, USA)) overnight. One hundred microliters of each overnight culture was then added to a separate Erlenmeyer flask containing 30 mL of TSB with antibiotics (25 µg/mL novobiocin and 20 µg/mL nalidixic acid) and cultured under the same conditions for 4 h. Only the 4 h cultures were used for infections. The optical density of the 4 h cultures were determined using a Molecular Devices Microplate Reader SpectraMax Plus (Molecular Devices, San Jose, CA, USA) at 600 nm endpoint absorbance. The measurement derived from a spectrophotometer was converted to bacteria count using the Agilent OD600 online calculator for *Escherichia coli*. To calculate the number of bacteria required for each assay, n (the required number of bacteria for each sample) was divided by bacteria OD600 (optical density measured at 600 nm converted to bacteria cell count) multiplied by 1000 (i.e., (n/OD600) × 1000).

### 2.3. Infection of Cells with Salmonella

Using HD11 cell counts, appropriate volumes of cell suspension to obtain 1 × 10^6^ cells were plated in 12- or 24-well VWR multiwell cell culture plates (VWR, Radnor, PA, USA) for approximately 2 h to adhere to the wells. These cells were then infected for 1 h with a multiplicity of infection (MOI) of 100 *Salmonella* per HD11 cell (100:1) for each serovar in three well replicates plus control. Infected cells were treated with 100 µg/mL of gentamicin (Sigma-Aldrich, St. Louis, MO, USA) for 30 min (to yield 1.5 h) or 1 h postinfection (p.i.) and incubated in gentamicin-free media for an additional 1 h for a total of 3 h p.i. Gentamicin is an antibiotic, targeting mostly Gram-positive bacteria [38]. However, studies have shown that treatment with gentamicin after *Salmonella* infection kills extracellular bacteria [38]. Therefore, treatment with gentamicin ensures that only changes due to intracellular *Salmonella* are being measured. The infected cells and control cells were used for experiments using different techniques as described below.

### 2.4. Gentamicin Protection Assay

One million cells were plated in 12- or 24-well VWR multiwell cell culture plates (VWR, Radnor, PA, USA) for approximately 2 h to adhere to the wells. These cells were then infected for 1 h with a multiplicity of infection (MOI) of 100:1 for each serovar in three well replicates plus control. Infected cells were treated with 100 µg/mL of gentamicin (Sigma-Aldrich, St. Louis, MO, USA) for 30 min (to yield 1.5 h) or 1 h postinfection (p.i.) and incubated in gentamicin-free media for an additional 1 h for a total of 3 h p.i. To evaluate the role of mTOR in *Salmonella* infections, 100 ng/mL final concentration of rapamycin (Sigma-Aldrich, St. Louis, MO, USA) or 2 μM of MHY1485 (Millipore Sigma, Burlington, MA, USA) was added to incubation media before infection.

After infection and treatment with gentamicin, the cells were lysed in 0.01 M of phosphate-buffered saline (PBS) (Sigma-Aldrich, St. Louis, MO, USA) containing 1% triton X100 (Sigma-Aldrich, St. Louis, MO, USA). After lysis, 100 µL from each well was added to 15 mL centrifuge tubes containing 9.9 mL of 0.01 M PBS (10^2^) and mixed gently. One thousand microliters of each 10^2^ mix was added to a separate 15 mL centrifuge tube containing 9 mL of PBS (10^3^) and mixed gently. One thousand microliters of 10^3^ was added to a centrifuge tube containing 9 mL of PBS, mixed gently, and plated in tryptic soy agar plates containing antibiotics (25 µg/mL novobiocin and 20 µg/mL nalidixic acid). After 12–18 h incubation at 37 °C, the bacteria colonies formed on the plates were counted and recorded as colony-forming units (CFUs).

### 2.5. Kinome Peptide Array Analysis

The kinome peptide array protocol performed for HD11 cells is briefly described below. The detailed protocol can be found in a publication by Arsenault et al. [39]. Cells were lysed in 100 µL of specially made lysis buffer containing protease inhibitors. The lysates were centrifuged, and 70 µL of the supernatant was mixed with 10 µL of activation mixture containing 500 µM of ATP to activate the kinases in the samples. Approximately 80 µL of each sample was applied to a glass peptide array (JPT Peptide Technologies, Berlin, Germany) and incubated in a sealed container placed in a 5% CO_2_ incubator at 37 °C for 2 h. After incubation, sample residues were washed off the arrays and the arrays were stained in phospho-specific fluorescent ProQ Diamond Phosphoprotein Stain (Life Technologies, Carlsbad, CA, USA) for 1 h. The arrays were submerged in a destain solution containing 20% acetonitrile (EMD Millipore Chemicals, Billerica, MA, USA) and 50 mM sodium acetate (Sigma-Aldrich, St. Louis, MO, USA) to remove non-phospho-specific binding. The arrays were scanned in a Tecan PowerScanner microarray scanner (Tecan Systems, San Jose, CA, USA) at 532 to 560 nm with a 580-nm filter to detect dye fluorescence.

The images of the scanned array were gridded manually to fit the phospho-specific spots and extract signal intensity using GenePix Pro software (version 7.2.29 1, Molecular Devices, CA, USA). Microsoft Excel 2016 (Redmond, WA) files containing kinomic data were generated and further analyzed using the online normalization and analysis tool known as Platform for Intelligent, Integrated Kinome Analysis (PIIKA2) [40]. The kinome peptide array data generated from PIIKA2 were analyzed by using other online databases like STRING [41], KEGG color and search pathway [42], UniProt [43,44,45], and PhosphosSitePlus [46].

The human UniProt accession and site information orthologous to chickens are used in the results and discussion of this paper because they are easily accessible. The chicken sites corresponding to their human identifiers used in this paper are reported in Appendix A. Appendix A also contains full names of all the proteins abbreviated in the result tables.

### 2.6. Seahorse XFp Metabolic Assay

The Seahorse XFp Analyzer (Agilent Technologies, Santa Clara, CA, USA) is technology that measures functional metabolic data, namely the extracellular acidification rate (ECAR) and oxygen consumption rate (OCR) [47,48]. The seahorse XFp analyzer was used to perform metabolic analysis of HD11 cells before and after infection (with *S.* Enteritidis or *S.* Heidelberg).

Cells were prepared for plating in a Seahorse mini culture plate (Agilent Technologies, Santa Clara, CA, USA) by adding 5 mL of trypsin to each T75 flask of cells. The cell–trypsin suspension was transferred to a 15 or 50 mL centrifuge tube. Each flask was washed with 5 mL of IMDM media. The washes were transferred into the cell–trypsin suspension tubes and centrifuged at 300 × *g* for 5 min. After centrifugation, the supernatant was discarded, and the cell pellet was collected and resuspended in 2–5 mL of IMDM media. The cells were counted to determine the volume of suspension needed to plate 50,000 cells per well. The desired volume of cells (50 µL of IMDM–cell mixture) was added to the wells of the mini culture plate (excluding wells A and H). The wells on the side of the plate were moated with 400 µL of double-deionized water. After the plating process, the plates were incubated in 5% CO_2_ for at least 2 h. It is strongly advised to calibrate the seahorse machine before each run; therefore, calibration plates were prepared at least 8 h before each experiment. The calibration plates were prepared by adding 400 µL of double-deionized water to the moat wells and 200 µL of Agilent Seahorse calibrant to the eight wells that held the cartridge. The cartridge was placed into the wells and incubated at 37 °C in a non-CO_2_ incubator. Bacteria serovars used for infection were prepared as described in the Section 2.2.

After 2–5 h of mini culture plate incubation at 37 °C with CO_2_, 200 µL of Seahorse media (XF DMEM medium, pH 7.4, with 5 mM HEPES (Agilent Technologies, Santa Clara, CA, USA)) containing 1% 200 mM glutamine and 1% 100 mM sodium pyruvate was added to each well (including blank wells A and H). After addition of seahorse media to the mini culture plates, the mini plates were incubated at 37 °C in a non-CO_2_ incubator for 30 min to 1 h. During this time, the calibration plate was removed from non-CO_2_ incubator and placed into the Seahorse XFp to calibrate the machine. After the machine was fully calibrated, bacteria were added to the designated wells of the mini culture plate. The calibration plate was then replaced by the mini culture plate with the infected cells to start the assay.

### 2.7. Statistics

For gentamicin protection assays, analysis of variance (ANOVA) and Tukey–Kramer post hoc tests were performed for all groups at the 30 min and 2 h p.i. timepoints, i.e., control (HD11 cells without infection or treatments), infected HD11 cells, HD11 cells with treatment only (rapamycin or MHY1485), and infected HD11 cells with treatments. For the Seahorse metabolic flux assays, analysis of variance (ANOVA) and Tukey–Kramer post hoc tests were performed for the control groups and infected cell groups at 30 min and 2 h p.i. Analysis of variance (ANOVA) and Tukey–Kramer post hoc tests were performed to compare within and amongst groups using JMP pro 14.0.0. For the kinome peptide array, a one-sided paired t-test between infected (treatment) and control values was performed for each peptide via PIIKA2 [40].

## 3. Results

### 3.1. Salmonella Alters Host (HD11 Cells) Immunometabolism

Analysis of the kinome peptide array performed on chicken macrophage-like cells infected with *S.* Enteritidis and *S.* Heidelberg showed significant changes in peptides phosphorylated in immune and metabolic pathways compared to control. A majority of these proteins belong to immunometabolic pathways, including the 5′ adenosine monophosphate-activated protein kinase (AMPK), hypoxia inducible factor (HIF), insulin, and mammalian target of rapamycin (mTOR) signaling pathways. Table 1 highlights the number of signaling proteins that are similarly altered in chicken macrophages infected with *S.* Enteritidis and *S.* Heidelberg at 30 min and 2 h p.i.

### 3.2. S. Enteritidis and S. Heidelberg Induce Differential Phosphorylation of Cytoskeletal Proteins

When *Salmonella* invades, it engages cellular responses and injects its proteins into the hosts’ cells; these proteins can alter host kinase activities [49,50,51]. To determine the different effects *S.* Enteritidis and *S.* Heidelberg each have on the kinase activity of cytoskeleton proteins in chicken macrophages, an in-depth analysis of the pathways observed in the kinome peptide array data (Table 1) was performed. The effector proteins produced by *Salmonella* can alter the cell cytoskeleton by affecting the activities of Rho proteins [52]. The kinome peptide array showed that there was an increase in phosphorylation of RhoA on its inhibitory site (S188) in *S.* Enteritidis infected cells (Table 2), inhibiting RhoA activity. For *S.* Heidelberg, there was a decrease in phosphorylation on this site. An increase in phosphorylation of the inhibitory site indicates inhibition of the ability of RhoA to drive the stabilization of cytoskeletal filaments [53], thus allowing bacteria to easily invade the cell [54]. There is also a decrease in phosphorylation of ROCK2 on its inhibitory site (Y722). ROCK is activated by binding to RhoA in its active GTP-bound state [55]; however, because RhoA is being inhibited in *S.* Enteritidis infected cells, RhoA downstream signaling to ROCK proteins should also be inhibited. Therefore, this phosphorylation could be due to other kinases.

### 3.3. S. Enteritidis and S. Heidelberg Induce Differential Phosphorylation of Metabolic Proteins to Promote Their Survival

Once in the gut, *Salmonella* is capable of initiating and utilizing host immunometabolic responses for its benefit [56,57]. It is hypothesized that *Salmonella* induces and promotes metabolic activities in the gut to promote its survival and growth [18,58,59]. Interestingly, the kinome peptide array revealed that these metabolic activities may be different for *S.* Enteritidis and *S.* Heidelberg. A greater increase in glycolytic protein activity was observed 30 min after *S.* Enteritidis infection than was observed in *S.* Heidelberg infected cells at this time. *S.* Enteritidis infected cells showed increased phosphorylation of both phosphofructokinases (PFK1 and PFK2), while *S.* Heidelberg infected cells did not (Table 3). Although the effects of phosphorylation are not known for many of these sites, we observed that *S.* Enteritidis infection initiated more glycolytic kinase activity than *S.* Heidelberg infection. At 30 min after *S.* Enteritidis infection, increased phosphorylation of the energy sensor protein AMPK on its active site (T183) was observed, indicating increased activity of AMPK (Table 3). *S.* Heidelberg infected cells showed the inhibition of AMPK via increased phosphorylation on its inhibitory site (S496). The increased activity of AMPK indicates a decrease in energy availability or decrease in the ratio of ATP to ADP/AMP [60]. This agrees with the observed increase in kinase activity of the glycolytic enzymes for *S.* Enteritidis at 30 min p.i. (Table 3).

Some of these metabolic changes are directly linked to immune or inflammatory responses. HIF-1α, which induces the transcription of inflammatory factors as well as genes involved in glucose metabolism, was increasingly phosphorylated on its inhibitory site (S247) in *S.* Heidelberg infected cells (Table 3). The decrease in glycolytic activity appeared unique to *S.* Heidelberg 30 min postinfection. Comparison of the changes in the phosphorylation of peptides on proteins involved in glycolysis of *S.* Heidelberg 30 min p.i. and *S.* Heidelberg 2 h p.i. revealed that kinase activity was not detected for phosphoglucose isomerase, PFK, and aldolase in the kinome peptide array data of *S.* Heidelberg 30 min p.i. However, there were kinase activities detected for the enzymes downstream of aldolase. There are instances where the products of phosphoglucose isomerase, PFK, and aldolase can be made available without the involvement of these three enzymes via an alternative substrate provider to continue the breakdown of glucose. The pentose phosphate pathway is an alternative substrate provider for glycolysis [61]. Specifically, undergoing the pentose phosphate pathway makes available fructose-6-phosphate and glyceraldehyde-3-phosphate [61], end products of the enzymes mentioned above. The pentose phosphate pathway also generates NADPH, which serves as a cofactor for inducible nitric oxide synthase (iNOS) for the production of nitric oxide (NO) [61,62]. This is supported in a publication by Haiqi et al. 2018, where the researchers observed a significant decrease in iNOS phosphorylation on its inhibitory site, thus inducing its activity [18]. The researchers also performed a nitrite assay, and the results showed a gradual increase in nitrite concentration which is comparable to NO generation in *S.* Heidelberg infected HD11 cells [18].

The increased activity of glycolytic proteins and the induction of glycolysis is an indication of proinflammation. Based on these results, *S.* Enteritidis infection induces more proinflammatory responses in chicken macrophages than *S.* Heidelberg. Besides the decrease in glycolytic activity in early *S.* Heidelberg infections, these results also indicate that *S.* Heidelberg may induce pentose phosphate pathway activity which may promote NO metabolism that is beneficial for bacteria growth and survival.

### 3.4. S. Enteritidis Infection Induces an Early Increase in Glucose Metabolism, and Early S. Heidelberg Infection Dampens Glucose Metabolism

**Figure 1:**
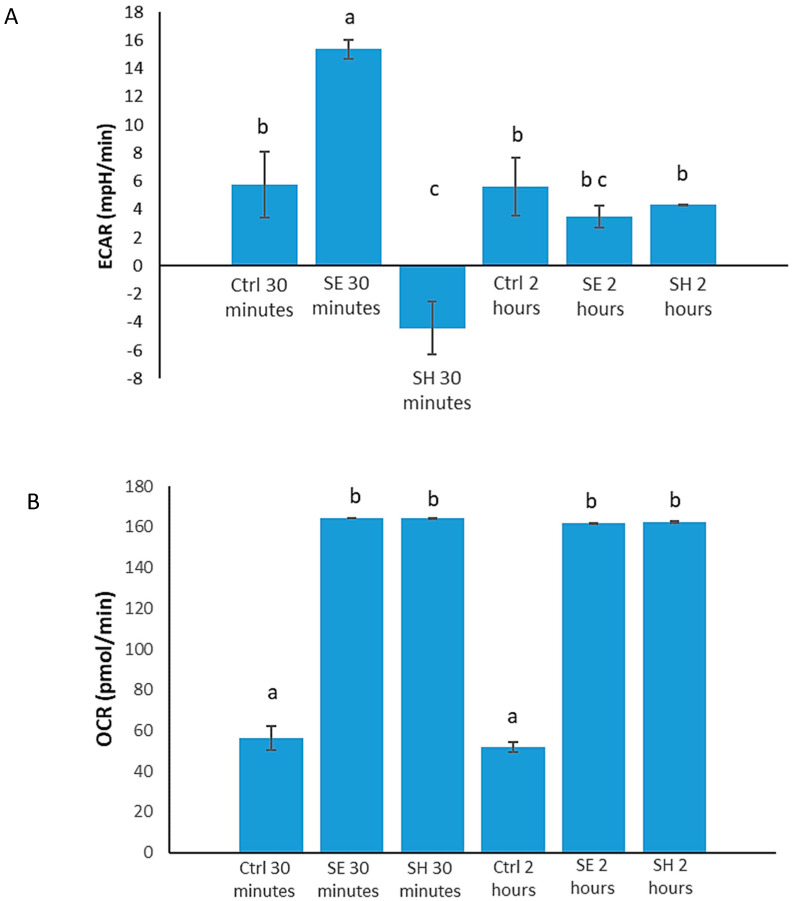
The metabolic differentiation induced by *S.* Enteritidis and *S.* Heidelberg during infection of chicken macrophages. (**A**) extracellular acidification rate (ECAR) readings and (**B**) and oxygen consumption rate (OCR) readings of *S.* Enteritidis and *S.* Heidelberg infected HD11 cells compared to uninfected HD11 cells. Before running the assay, the cells plated in a mini culture plate were incubated in a CO_2_-free incubator for at least 30 min upon the addition of glucose-free media. After incubation, *S.* Enteritidis or *S.* Heidelberg was added to the respective wells and the assay was started. Bars with the same letters on the top are not significantly different from each other. *p* ≤ 0.05 observed using Tukey–Kramer statistical tests following ANOVA. Ctrl, Control; SE, *S.* Enteritidis; SH, *S.* Heidelberg.

Increased glycolysis is an indication of proinflammatory responses [24,63]. The kinome peptide array results showed an increase in glycolytic activities for *S.* Enteritidis and a reduction in glycolytic activities for *S.* Heidelberg. To determine changes in the metabolic states of HD11 cells during *S.* Enteritidis or *S.* Heidelberg infections at 30 min and 2 h p.i., a gentamicin-free metabolic flux assay measuring ECAR was performed on HD11 cells infected with each serovar. *S.* Enteritidis infected cells at 30 min p.i. showed an increase in ECAR (15.365 mpH/min) followed by a larger decrease (3.5 mpH/min) at 2 h postinfection (Figure 1A). ECAR measurements indicate the rate of glycolysis; thus, *S.* Enteritidis at 30 min postinfection increases glucose metabolism. Results from the *S.* Heidelberg infected cells showed a decrease in ECAR at 30 min p.i. (−4.41 mpH/min) compared to control (5.77 mpH/min), with a *p*-value of 0.02 (Figure 1A). At 2 h postinfection, there was a significant increase in ECAR readings of *S.* Heidelberg infected cells (4.31 mpH/min) compared to the 30 min results (Figure 1A). Thus, *S.* Heidelberg at 30 min postinfection induces a decrease in glucose metabolism in HD11 cells.

The metabolic assay also showed that *Salmonella* infections induce increased oxygen metabolism in HD11 cells. OCR measurements indicating the oxygen consumption of the HD11 cells showed an increase in OCR in cells infected with *S.* Enteritidis (164.525 pmol/min) and *S.* Heidelberg (164.19 pmol/min) at 30 min compared to control (56.44 pmol/min) (Figure 1B). This increase in OCR was sustained past the 2 h timepoint, with the OCR of *S.* Enteritidis infected cells being 165 pmol/min and that of *S.* Heidelberg infected cells being 165 pmol/min; meanwhile, the OCR of the control cells remained relatively low at 51.83 pmol/min (Figure 1B). These measurements comparing the OCR of infected cells to that of control cells showed statistical significance at both timepoints (*p* ≤ 0.0001), indicating that increased oxygen metabolism may be common to both serovars and *Salmonella* in general.

### 3.5. S. Enteritidis and S. Heidelberg Induce Differential Phosphorylation of Inflammatory Proteins

After invading host cells using the type III secretion system, *Salmonella* effector proteins also stimulate the activation of the NLRC4 inflammasome to induce pyroptosis to invade other cells [64]. *S.* Enteritidis infected cells showed early induction of caspase-1 activity via the increased phosphorylation on site S227. However, there was decreased phosphorylation of S227 for *S.* Enteritidis infected cells at 2 h p.i., while no significant data supported the phosphorylation of caspase-1 in *S.* Heidelberg infected cells. Caspase-1 is the final protein stimulated in the NLRC4 inflammasome to induce proinflammatory responses like pyroptosis. Therefore, its kinase activity in *S.* Enteritidis at 30 min p.i. suggests induction of its activity. Although kinase activities of other proteins involved in the NLRC4 inflammasome were observed in the kinome peptide array data of *S.* Heidelberg infected cells, there is no evidence to support caspase-1 activity.

Besides signs of pyroptosis, kinome data from early *S.* Enteritidis infection also showed stimulation of cell death signaling via the tumor necrosis factor (TNF) receptor-associated factor and proteins downstream of the receptor including Jun N-terminal kinase 1 (JNK1) and mitogen-activated protein kinase (MAPK)-interacting kinase 1 (MNK1). These signs were observed in chicken macrophages 30 min p.i. with *S.* Enteritidis and 2 h p.i. with *S.* Heidelberg. In detail, JNK1 (which is known to induce apoptosis [65,66]) was phosphorylated at site T183 (Table 4), thus stimulating early cell death in *S.* Enteritidis infected cells. MNK, which is also downstream of TNF-alpha–MAPK signaling was significantly less phosphorylated (Table 4) than control for *S.* Enteritidis at 30 min and *S.* Heidelberg at 2 h p.i. on the site T255, responsible for the inhibition of apoptosis [66]. In addition, the NFkB inhibitor IkB-alpha was also significantly more phosphorylated on its active site (Y42) in *S.* Heidelberg at 30 min p.i. (Table 4). Y42 also plays a role in the inhibition of apoptosis [67].

The kinome peptide array data also showed the decreased phosphorylation of the proapoptotic factor caspase-3 on its inhibitory site for both serovars at 30 min p.i. (Table 4). These changes in phosphorylation to positively affect apoptosis were commonly observed in *S.* Heidelberg at 2 h p.i. Thus far, the kinome peptide array data suggest that cell death is reduced in the initial stages of *S.* Heidelberg infections compared to the initial stages of *S.* Enteritidis infections. The changes in phosphorylation of toll-like receptors (TLRs) and cytokine receptors such as interleukin (IL)-6 indicate inflammatory stress. Moreover, *S.* Enteritidis may pose a significant challenge to the host cells during initial infection which results in the stimulation of programmed cell death early on. Meanwhile, apoptosis is not induced during the early stages of *S.* Heidelberg infection because less inflammation is induced in the host upon initial infection.

The energetic demands of these infections can affect the hosts’ immune capacity by suppressing activity of immunometabolic proteins like mTOR [68]. As energy demand increases, AMPK is activated, and this leads to the deactivation or inhibition of mTOR by AMPK [68]. mTOR is an immunometabolic protein that regulates the translation and synthesis of proteins, including those involved in immune responses [69,70]. Activity of mTOR was not detected via phosphorylation of mTOR on its active site S2448 in both *S.* Enteritidis and *S.* Heidelberg infected cells early on. Active mTORc1 phosphorylates 4EBP1 and S6K to promote protein synthesis [36,70] and cell growth and survival [36], respectively. At 2 h after *S.* Enteritidis and *S.* Heidelberg infection, mTOR is increasingly phosphorylated on its active site S2448 to induce activity in the cells. The initial absence of mTOR activity could mean that *Salmonella* targets mTOR for invasion or this is just a cellular response to prioritize cellular activities during infection.

### 3.6. S. Enteritidis Is More Invasive Than S. Heidelberg

**Figure 2:**
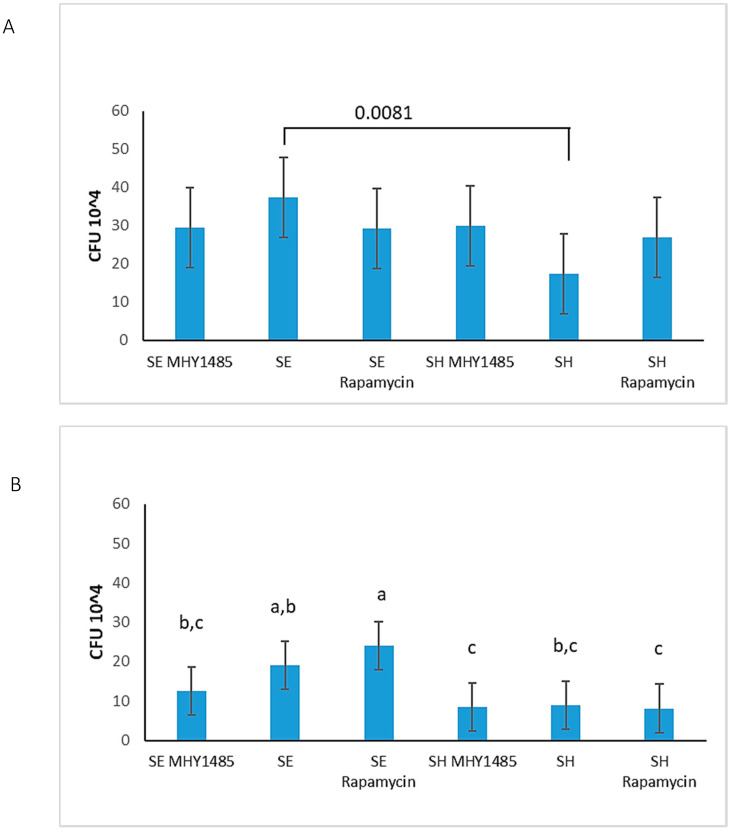
Invasiveness of *S.* Enteritidis (SE) and *S.* Heidelberg (SH) in HD11 macrophages at (**A**) 30 min postinfection and (**B**) 2 h postinfection. Each bar in this graph represents the average colony count or colony-forming units (CFUs) of respective serovars of *Salmonella* that infected HD11 cells treated with or without MHY1485 or rapamycin before 30 min gentamicin protection assay as shown in (**A**) (*p*-value indicated on graph) or before 1 h gentamicin protection assay as shown in (**B**). *p* ≤ 0.05 observed using ANOVA and Tukey–Kramer statistical tests. Bars not connected by the same letter are significantly different. *p* ≤ 0.05 observed using ANOVA and Tukey–Kramer statistical tests. Ctrl, Control; SE, *S.* Enteritidis; SH, *S.* Heidelberg.

The kinome peptide array data showed changes in the phosphorylation of cytoskeletal proteins that are more favorable to invasion by *S.* Enteritidis than *S.* Heidelberg (Table 2). Therefore, gentamicin protection assays were performed to quantify the ability of *S.* Enteritidis and *S.* Heidelberg to invade HD11 cells at 30 min and 2 h p.i. The kinome peptide array data also showed changes in mTOR activity in both serovars at different timepoints (Table 4). To determine the role of mTOR in *Salmonella* invasions, the cells were treated with the mTOR inhibitor rapamycin and the mTOR activator MHY1485 before infection, and gentamicin protection assays were performed. Results of the gentamicin protection assays showed that there was a significant increase in invasion by *S.* Enteritidis, as shown by the colony-forming unit (CFU) count, compared to *S.* Heidelberg at 30 min p.i. (Figure 2A). Meanwhile, at 2 h p.i., *S.* Enteritidis had higher plate counts than *S.* Heidelberg; however, this difference was not significant (Figure 2B). The overnight colony count for *S.* Enteritidis at 30 min was 37.44 × 10^4^, while that of *S.* Heidelberg at 30 min was 17.44 × 10^4^ (*p*-value of 0.0081). Thus, *S.* Enteritidis is better at invading HD11 cells compared to *S.* Heidelberg at 30 min p.i. There was a trend observed at 2 h after *S.* Enteritidis infection only (19 × 10^4^ CFUs) that showed an increase in plate counts in *S.* Enteritidis infection with rapamycin treatment (24 × 10^4^ CFUs) and a decrease in *S.* Enteritidis with MHY1485 treatment (12 × 10^4^ CFUs) (Figure 2B). However, the statistical analysis for these plate counts showed no significant difference between *S.* Enteritidis and its treatment groups at 2 h p.i. (Figure 2B). There were also no significant differences between *S.* Heidelberg and its treatment groups at 2 h p.i. (Figure 2B).

## 4. Discussion

In this study, we demonstrated the immunometabolic difference between *S.* Enteritidis and *S.* Heidelberg infections in chicken HD11 macrophages (Table 5). Although *S.* Enteritidis and *S.* Heidelberg are serovars belonging to the same subspecies of *Salmonella*, we observed that each induce different changes in energy metabolism and display different invasiveness in these macrophages. For successful survival in the host, *Salmonella* must first invade the host cell. The kinome peptide array data suggested that *S.* Enteritidis may be more efficient at invading the host cell than *S.* Heidelberg (Table 5) by inhibiting the activity of RhoA (Table 2) which activates ROCK2, responsible for regulating the cell cytoskeletal and actin filament stabilization [71]. Although the effects of phosphorylation for the sites of RGH06 and RGH17 are unknown (Table 2), these two proteins are known to inactivate GTPase by converting it to a GDP-bound state [72]. With the decrease in RHOA and ROCK2 activity, *S.* Enteritidis can easily affect other host cytoskeletal proteins using effectors from its T3SS to invade cells. This efficient invasiveness of *S.* Enteritidis over *S.* Heidelberg was supported by the gentamicin assay (Figure 2) that showed a greater average CFU count for *S.* Enteritidis in HD11 cells at 30 min p.i.

Moreover, we also observed that different immunometabolic activities are induced in *S.* Enteritidis and *S.* Heidelberg infections upon invasion. The metabolic activities observed in both serovars are crucial components of immune responses needed to clear infections, but studies have shown that *Salmonella* can use some host inflammatory and metabolic activities to create a suitable niche for survival [58,59]. Early *S.* Enteritidis infection induced an increase in glycolysis (Table 3, Figure 1) known to be a trademark of a proinflammatory response [24,63], while no signs of increased glycolytic activities were observed in HD11 cells infected with *S.* Heidelberg until the 2 h p.i. timepoint (Table 3, Figure 1). The kinome peptide array data suggested that the pentose phosphate pathway may have been induced in *S.* Heidelberg infected cells during initial infection (Table 3). The purpose of this response in the cell is not fully understood; however, it may be that the decrease in glycolysis is to dampen the inflammation induced during glucose metabolism and increase the production of NO via the pentose phosphate route [61,62]. The significance of this action is that it converts NO to nitrate, which *Salmonella* bacteria are known to metabolize for growth and survival [73]. Results from Haiqi et al. (2018) showed that *S.* Heidelberg infections induce significantly more NO than *S.* Enteritidis [18], yet further testing is required to validate the higher rate of the pentose phosphate pathway in *S.* Heidelberg infected cells than in those infected by *S.* Enteritidis to fully support this hypothesis. In short, these results revealed that *S.* Enteritidis and *S.* Heidelberg each induce different metabolic activities (Table 5) that influence the immune responses of the host cells via the AMPK, HIF, mTOR, and insulin pathways. We also demonstrated that *S.* Enteritidis and *S.* Heidelberg have similar effects on oxygen metabolism (Table 1, Table 5, and Figure 1B).

Studies have shown that *Salmonella* induces virulent mechanisms in host cells to stimulate cell death [59,74]. In this study, we identified changes in phosphorylation of programmed cell death inducing proteins during *S.* Enteritidis and *S.* Heidelberg infections. Caspase-3 and caspase-8 are both involved in the initiation of programmed cell death. S150 of caspase-3 is a site that has been found to inhibit apoptosis [75]. In the kinome peptide array dataset, we observed a decrease in phosphorylation of this inhibitory site (Table 4), which indicates a positive regulation of apoptosis. The same can be said for caspase-8 site S347 [75] (Table 4). Besides the decreased inhibition of caspase-3 at 30 min after *S.* Heidelberg infection, there was not much evidence in the kinome peptide array results supporting the occurrence of increased cell death in HD11 cells at 30 min p.i. However, at the *S.* Heidelberg 2 h p.i. point, we observed an increase in positive regulation of apoptotic factors via the decreased activity of proteins involved in the inhibition of apoptosis, such as IkB-alpha (Y42), MNK1 (T255), caspase-3, and caspase-8, and the increased activity of proapoptotic factors like JNK1 and IRF1 (Table 4). *S.* Enteritidis induces changes in the phosphorylation of the proinflammatory and pyroptosis-inducing protein caspase-1 (Table 4). *S.* Enteritidis also induced changes in phosphorylation of other proteins involved in the signaling of the NLRC4 inflammasome. This sign of inflammatory cell death, considered along with the changes in phosphorylation of JNK1 and caspase-3 and the absence of kinases that regulate cell death inhibitors like IkB-alpha and MNK at 30 min p.i. (Table 4), suggests that *S.* Enteritidis may cause more cell death than *S.* Heidelberg during initial infection (Table 5). Further testing to measure cell death is required before accepting this hypothesis.

Moreover, the inflammatory stress induced by *S.* Enteritidis and *S.* Heidelberg via the changes in phosphorylation of TLR and IL-6R (Table 4) may also play a role in cell death and invasion [76]. The gentamicin protection assays showed a significant difference between the invasiveness of *S.* Enteritidis and *S.* Heidelberg at 30 min p.i. (Figure 2A). At 2 h p.i., although not significant, *S.* Enteritidis plate counts were also higher than *S.* Heidelberg (Figure 2B). The high number of intracellular *S.* Enteritidis in HD11 macrophages highlights its ability to invade and form a suitable niche in the host. *S.* Enteritidis invades cells, causing a change to the M1 profile. This induces the proinflammatory response [24], including the expression of proinflammatory cytokines [77] and increased downstream and feedback activity of pathogen recognition receptors (PRR) like TLR [76]. The ability of *S.* Enteritidis to invade more host cells increases due to this increase in proinflammatory factors [78]. This increased inflammation can ultimately lead to cell death and rupture, enabling the bacteria to invade neighboring healthy cells (Table 5).

Although apoptosis is a naturally occurring noninflammatory process carried out in a cell, it is also a mechanism to clear infected cells. *Salmonella* uses this response to its advantage. Apoptotic bodies formed from infected cells contain vacuoles of *Salmonella*, causing phagocytic cells to become infected as a result of ingesting such bodies [23,79]. Thus, induction of programmed cell death early on would enable *S.* Enteritidis to carry out cellular invasion. The colony plate count for *S.* Heidelberg remained low throughout both timepoints, yet *S.* Heidelberg is still capable of inducing substantial changes in the immune and metabolic signaling of host cells. This implies that *S.* Heidelberg does not require a large-scale invasion to survive and create a suitable niche in host cells.

These inflammatory responses can be energetically draining and may affect other processes in host cells [60,68]. mTOR complex 1 has been of major interest in the study of host response to bacterial infections because of its role in regulation and synthesis of proteins, many of which are involved in immune regulation [36,70,80]. The kinome peptide array showed a lack of mTORC1 kinase activity specifically at the active site S2448 at 30 min p.i. but showed an increase in the phosphorylation of this site at 2 h p.i. for both *S.* Enteritidis and *S.* Heidelberg. The role of mTOR in cell survival and protein synthesis raises questions about its involvement in bacterial invasion and survival during infection. To determine whether inhibition or activation of mTOR would increase or decrease *Salmonella* invasiveness, HD11 cells were treated with 100 g/mL of rapamycin or 2 µM of MHY1485 before *Salmonella* infection. The results showed no difference in invasion of HD11 macrophages treated with MHY1485 and infected by *S.* Enteritidis or *S.* Heidelberg at 30 min or 2 h p.i. (Figure 2). Although MHY1485 showed a decrease in *S.* Enteritidis count at 2 h and rapamycin showed an increase in *S.* Enteritidis count at 2 h, statistical analysis showed no significant difference between the treatment groups and *S.* Enteritidis infection alone (Figure 2). Comparing *S.* Enteritidis 2 h p.i. MHY1485 and rapamycin treatment groups to each other showed that increasing mTOR activation over time may improve clearance of the bacteria compared to decreasing or inhibiting mTOR (Table 5, Figure 2B).

## 5. Conclusions

In summary, *S.* Enteritidis and *S.* Heidelberg induce different changes in the phosphorylation of immunometabolic signaling peptides compared to control in vitro. As shown by the kinome peptide array, there are key differences in phosphorylation of peptides on proteins involved in energy metabolism, protein regulation, apoptosis, cytoskeletal regulation, and inflammation. These proteins include PFK 1 and 2; AMPK; caspases 1, 3, and 8; HIF-1α; TLR; RhoA; and more. These findings were further validated using the metabolic flux assays and gentamicin protection assays. The metabolic flux assays which measured ECAR and OCR demonstrated that (i) *S.* Enteritidis at 30 min p.i. resulted in increased glucose metabolism, (ii) *S.* Heidelberg at 30 min p.i. resulted in decreased glucose metabolism, and (iii) both *Salmonella* serovar infections induce increased oxygen metabolism compared to control. Gentamicin protection assays performed at 30 min and 2 h postinfection revealed that *S.* Enteritidis bacteria are more invasive than *S.* Heidelberg.

Overall, these results support the observations of invasiveness of *S.* Enteritidis and persistence of *S.* Heidelberg in poultry and an understanding of the immunometabolic activities that may contribute to such differences. That is, the immunometabolic responses that *S.* Enteritidis exploits in hosts for increased invasion only present short-term benefits to the bacteria. Meanwhile, the delayed host immunometabolic response to *S.* Heidelberg, at the cost of decreased invasiveness, offers the long-term benefits of increased persistence to the bacteria. The tradeoff for *S.* Enteritidis in increasing its invasiveness is the increased immune response produced by the host to clear the infection, hence the decrease in prevalence of *S.* Enteritidis. The persistence of *S.* Heidelberg infection is evident in the increasing isolation and prevalence of this bacteria in poultry over *S.* Enteritidis in the past decade. This project reveals the difficulty associated with efficiently treating *Salmonella* infections because different serovars of *Salmonella* may induce different immunometabolic responses in hosts. Therefore, an immune or metabolic target for the treatment of one serovar may benefit another serovar.

## Figures and Tables

**Table 1:** *Salmonella* alters the phosphorylation of proteins in key immunometabolic pathways.

Signal Transduction Pathways	Proteins altered 30 min p.i.	Proteins altered 2 h p.i.
Insulin signaling	17	23
AMPK signaling	21	19
mTOR signaling	13	15
HIF-1α signaling	16	21

The number of proteins that showed the same changes in phosphorylation (increased or decreased phosphorylation) in immunometabolic signal transduction pathways for both *S.* Enteritidis and *S.* Heidelberg infected HD11. This information was derived from the STRING database’s [41] top 20 KEGG pathway [42] output of the kinome peptide array data.

**Table 2:** Phosphorylation changes in major cytoskeletal proteins during *S.* Enteritidis and *S.* Heidelberg infection of chicken macrophages.

UniProt Accession	Protein Name	Site/Effect of Phosphorylation	Phosphorylation Change 30 Min		Phosphorylation Change 2 h	
			*S.* Enteritidis	*S.* Heidelberg	*S.* Enteritidis	*S.* Heidelberg
P61586	RhoA	S188/activity inhibited	↑	↓	↑	↓
O75116	ROCK2	Y722/activity inhibited	↓	∅	↓	↓
O43182	RGH06	Y407/unspecified	↓	∅	∅	↓
Q68EM7	RGH17	S484/unspecified	↑	↑	↑	↑

The changes in the phosphorylation states of key proteins in cell cytoskeletal control and stability as shown in the kinome peptide array data. The UniProt accession and site information belong to human proteins that are orthologues of chicken proteins and were collected from the PhosphoSite and UniProt databases. The orthologous chicken phosphorylation sites are reported in Appendix A. The arrows represent the sites discussed or considered for the respective proteins mentioned. ↑, significantly more phosphorylated and consequence of phosphorylation unknown; ↑, significantly more phosphorylated on an inhibitory site; ↓, significantly less phosphorylated and consequence of phosphorylation unknown; ↓, significantly less phosphorylated on an inhibitory site; ∅, not significant in the indicated dataset.

**Table 3:** Changes in the phosphorylation of immunometabolic peptides of *S.* Enteritidis and *S.* Heidelberg infected chicken macrophages.

Protein/Peptide	Phosphorylation Change 30 Min		Phosphorylation Change 2 h	
	*S.* Enteritidis	*S.* Heidelberg	*S.* Enteritidis	*S.* Heidelberg
PFK1	↑↓	↓	↑↑↑↓	↑↑↑↓
PFK2	↑↑(S461)	∅	↑↑(S461)	↑↑(S461)
GPI	↓(S185)	↓(S185)	∅	↑↑
PhK	↑↓	↑	↓↓↓	↑↓
GAPDH	↑↑	↑	∅	↑↓
PGK	↑	↑	↓	↓
PGM	↓	↓	↑↓↓	↓↓
PKM	↑	↑↑↑	↑↑	↑↑↓
AMPK	↑↑↑(T183)↓↓	↑↑↑(S496)↓↓↓	↓↓	↑↑↑↑(S496)↓↓
HIF-1α	↑↓(S247)	↑↑(S247)	↑(S247)	↑(S247)↓
S6K	↓↓	↓	↑↓	↑
4EBP1	↑	↓↓	↓↓↓	↓↓

The changes in the phosphorylation states of immune- and metabolic-related peptides involved in AMPK, mTOR, and HIF-1α signaling as shown in the kinome peptide array data. The arrows represent significant changes in phosphorylation at different kinase target sites. ↑, significantly more phosphorylated and consequence of phosphorylation unknown; ↑, significantly more phosphorylated on an active site; ↑, significantly more phosphorylated on an inhibitory site; ↓, significantly less phosphorylated and consequence of phosphorylation unknown; ↓, significantly less phosphorylated on an active site; ↓, significantly less phosphorylated on an inhibitory site; ∅, not significant in the indicated dataset. PhK, phosphorylase kinase; GAPDH, glyceraldehyde-3-phosphate dehydrogenase; PGK, phosphoglycerate kinase 1; PGM, phosphoglucomutase; S6K, ribosomal protein S6 kinase beta-1; 4EBP1, eukaryotic translation initiation factor 4E-binding protein 1.

**Table 4:** Phosphorylation changes in major immune and immunoregulatory proteins during *S.* Enteritidis and *S.* Heidelberg infection of chicken macrophages.

UniProt Accession	Protein Name	Site/Effect of Phosphorylation	Phosphorylation Change 30 Min		Phosphorylation Change 2 h	
			*S.* Enteritidis	*S.* Heidelberg	*S.* Enteritidis	*S.* Heidelberg
P29466	Caspase-1	S227/unspecified	↑	∅	↓	∅
Q96P20	NLRP3	T233/unspecified	↓	↑	∅	↓
P10914	IRF1	Y109/unspecified	∅	∅	↑	↑
Q9BXL7	CARD11	S116/unspecified	↑	↑	↓	↓
O60602	TLR5	Y798/unspecified	∅	↑	↓	↓
O15455	TLR3	Y858/unspecified	↓	∅	↑	↓
P40189	IL-6R	S782/unspecified	↑	↑	↓	∅
P42574	Caspase-3	S150/activity inhibited	↓	↓	∅	↓
Q14790	Caspase-8	S347/activity inhibited	∅	∅	↓	↓
Q9BUB5	MNK	T255/activity induced	∅	↓	∅	↓
P45983	JNK1	T183/unspecified	↑	∅	∅	↑
P25963	IkB-alpha	Y42/activity induced	∅	↑	↓	∅
P42345	mTOR	S2448/activity induced	∅	∅	↑	↑

The changes in the phosphorylation states of some key proteins involved in immune signaling and immune regulation as shown in the kinome peptide array data. The UniProt accession and site information belong to human proteins that are orthologues of chicken proteins and were collected from the PhosphoSite and UniProt databases. The orthologous chicken phosphorylation sites are reported in Appendix A. The arrows represent the sites discussed or considered for the respective proteins mentioned. ↑, significantly more phosphorylated and consequence of phosphorylation unknown; ↑, significantly more phosphorylated on an inhibitory site; ↓, significantly less phosphorylated and consequence of phosphorylation unknown; ↓, significantly less phosphorylated on an inhibitory site; ↑, significantly more phosphorylated on an active site; ↓, significantly less phosphorylated on an active site; ∅, not significant in the indicated dataset.

**Table 5:** Summary of chicken macrophage responses to infections by *S.* Enteritidis and *S.* Heidelberg.

*S.* Enteritidis 30 min p.i.	*S.* Heidelberg 30 min p.i.	*S.* Enteritidis 2 h p.i.	*S.* Heidelberg 2 h p.i.
Increased rate of glycolysis and no significant change in pentose phosphate pathway activity	Decreased rate of glycolysis and increased pentose phosphate pathway activity	Decreased rate of glycolysis and no significant change in pentose phosphate pathway activity	Increased rate of glycolysis and maintained increased pentose phosphate pathway activity
Increased invasiveness and increased cell death *	No change in invasiveness and cell death	No change in invasiveness and decreased cell death	No changes in invasiveness and increased cell death
Increased rate of oxygen consumption	Increased rate of oxygen consumption	Increased rate of oxygen consumption	Increased rate of oxygen consumption
No response to mTOR treatments	No response to mTOR treatments	Response to mTOR treatments	No response to mTOR treatments

Summary of chicken macrophage responses to *S.* Enteritidis infections and *S.* Heidelberg infections at 30 min p.i. and 2 h p.i. This summary is based on the results of the kinome peptide array analysis, metabolic assays, and gentamicin protection assays. Pentose phosphate pathway activity reported in this summary is supported by He et al. [18]. * denotes evidence of inflammatory cell death.

## Appendix A

**Table A1:** Protein names and chicken phosphorylation sites corresponding to the human sites reported in this paper.

Protein Name	Human UniProt Accession	Human Site	Chicken Site
Transforming protein RhoA (RhoA)	P61586	S188	S199
Rho-associated, coiled-coil-containing protein kinase 2 (ROCK2)	O75116	Y722	Y507
Rho GTPase-activating protein 6	O43182	Y407	Y211
Rho GTPase-activating protein 17	Q68EM7	S484	S479
Caspase-1	P29466	S227	S106
NACHT, LRR, and PYD domains-containing protein 3 (NLRP3)	Q96P20	T233	T24
Interferon regulatory factor 1 (IRF-1)	P10914	Y109	Y109
Caspase recruitment domain-containing protein 11 (CARD11)	Q9BXL7	S116	S118
Toll-like receptor 5 (TLR5)	O60602	Y798	Y800
Toll-like receptor 3 (TLR3)	O15455	Y858	Y854
Interleukin-6 receptor subunit beta (IL-6R)	P40189	S782	S757
Caspase-3	P42574	S150	S158
Caspase-8	Q14790	S347	S350
MAP kinase-interacting protein kinase 1 (MNK1)	Q9BUB5	T255	T199
Jun N-terminal kinase 1 (JNK1)	P45983	T183	T183
NF-kappa-B inhibitor alpha/I-kappa-B-alpha (IkB-α)	P25963	Y42	Y46
Serine/threonine-protein kinase mTOR (mTOR)	P42345	S2448	S2352
6-Phosphofructo-2-kinase/fructose-2,6-bisphosphatase 3 (PFK2)	Q16875	S461	S462
Glucose-6-phosphate isomerase (GPI)	P06744	S185	S184
5′-AMP-activated protein kinase catalytic subunit α-1 (AMPK)	Q13131	T183	T185
Hypoxia-inducible factor 1-alpha (HIF-1α)	Q16665	S247	S247

## References

[1] Andino A., Hanning I. (2015). Salmonella enterica: Survival, Colonization, and Virulence Differences among Serovars. Sci. World J..

[2] Swaggerty C., Kogut M.H., He H., Genovese K.J., Johnson C., Arsenault R.J. (2017). Differential Levels of Cecal Colonization by Salmonella Enteritidis in Chickens Triggers Distinct Immune Kinome Profiles. Front. Vet. Sci..

[3] Majowicz S.E., Musto J., Scallan E., Angulo F.J., Kirk M.D., O’Brien S., Jones T.F., Fazil A., Hoekstra R.M. (2010). The Global Burden of Nontyphoidal Salmonella Gastroenteritis. Clin. Infect. Dis..

[4] Gal-Mor O., Boyle E.C., Grassl G.A. (2014). Same species, different diseases: How and why typhoidal and non-typhoidal Salmonella enterica serovars differ. Front. Microbiol..

[5] Feasey N.A., Dougan G., Kingsley R.A., Heyderman R.S., Gordon M.A. (2012). Invasive non-typhoidal salmonella disease: An emerging and neglected tropical disease in Africa. Lancet.

[6] Porwollik S., Boyd E.F., Choy C., Cheng P., Florea L., Proctor E., McClelland M. (2004). Characterization of Salmonella enterica Subspecies I Genovars by Use of Microarrays. J. Bacteriol..

[7] Demczuk W., Soule G., Clark C., Ackermann H.-W., Easy R., Khakhria R., Rodgers F., Ahmed R. (2003). Phage-Based Typing Scheme for Salmonella enterica Serovar Heidelberg, a Causative Agent of Food Poisonings in Canada. J. Clin. Microbiol..

[8] Haeusler G.M., Curtis N. (2013). Non-typhoidal Salmonella in children: Microbiology, epidemiology, and treatment. Advances in Experimental Medicine and Biology.

[9] Nair D.V.T., Venkitanarayanan K., Johny A.K. (2018). Antibiotic-Resistant Salmonella in the Food Supply and the Potential Role of Antibiotic Alternatives for Control. Foods.

[10] Scallan E., Hoekstra R.M., Angulo F.J., Tauxe R.V., Widdowson M.-A., Roy S.L., Jones J.L., Griffin P.M. (2011). Foodborne Illness Acquired in the United States—Major Pathogens. Emerg. Infect. Dis..

[11] Gordon M.A. (2008). Salmonella infections in immunocompromised adults. J. Infect..

[12] Shimoni Z., Pitlik S., Leibovici L., Samra Z., Konigsberger H., Drucker M., Agmon V., Ashkenazi S., Weinberger M. (1999). Nontyphoid Salmonella Bacteremia: Age-Related Differences in Clinical Presentation, Bacteriology, and Outcome. Clin. Infect. Dis..

[13] Zhao X., Gao Y., Ye C., Yang L., Wang T., Chang W. (2016). Prevalence and Characteristics of Salmonella Isolated from Free-Range Chickens in Shandong Province, China. BioMed Res. Int..

[14] Andres V.M., Davies R.H. (2015). Biosecurity Measures to Control Salmonella and Other Infectious Agents in Pig Farms: A Review. Compr. Rev. Food Sci. Food Saf..

[15] Shah D.H., Paul N.C., Sischo W.C., Crespo R., Guard J. (2017). Population dynamics and antimicrobial resistance of the most prevalent poultry-associated Salmonella serotypes. Poult. Sci..

[16] Olsen S.J., Bishop R., Brenner F.W., Roels T.H., Bean N., Tauxe R.V., Slutsker L. (2001). The Changing Epidemiology of Salmonella: Trends in Serotypes Isolated from Humans in the United States, 1987–1997. J. Infect. Dis..

[17] Djeffal S., Mamache B., Elgroud R., Hireche S., Bouaziz O. (2018). Prevalence and risk factors for Salmonella spp. contamination in broiler chicken farms and slaughterhouses in the northeast of Algeria. Vet. World.

[18] He H., Arsenault R.J., Genovese K.J., Johnson C., Kogut M.H. (2018). Chicken macrophages infected with Salmonella (*S.*) Enteritidis or *S.* Heidelberg produce differential responses in immune and metabolic signaling pathways. Vet. Immunol. Immunopathol..

[19] Ciraci C., Tuggle C.K., Wannemuehler M.J., Nettleton D.S., Lamont S.J. (2010). Unique genome-wide transcriptome profiles of chicken macrophages exposed to Salmonella-derived endotoxin. BMC Genom..

[20] Jarvis N.A., Donaldson J.R., O’Bryan C.A., Ricke S.C., Crandall P.G. (2017). Listeria monocytogenes infection of HD11, chicken macrophage-like cells. Poult. Sci..

[21] Beug H., von Kirchbach A., Doderlein G., Conscience J.-F., Graf T. (1979). Chicken hematopoietic cells transformed by seven strains of defective avian leukemia viruses display three distinct phenotypes of differentiation. Cell.

[22] Wisner A.L.S., Potter A.A., Köster W. (2011). Effect of the Salmonella Pathogenicity Island 2 Type III Secretion System on Salmonella Survival in Activated Chicken Macrophage-Like HD11 Cells. PLoS ONE.

[23] Hirayama D., Iida T., Nakase H. (2017). The Phagocytic Function of Macrophage-Enforcing Innate Immunity and Tissue Homeostasis. Int. J. Mol. Sci..

[24] Langston P.K., Shibata M., Horng T. (2017). Metabolism Supports Macrophage Activation. Front. Immunol..

[25] Gog J.R., Murcia A., Osterman N., Restif O., McKinley T.J., Sheppard M., Achouri S., Wei B., Mastroeni P., Wood J.L.N. (2012). Dynamics of Salmonella infection of macrophages at the single cell level. J. R. Soc. Interface.

[26] Gorbach S.L., Baron S. (1996). Microbiology of the Gastrointestinal Tract. Medical Microbiology.

[27] Yang M., Xu J., Wang Q., Zhang A., Wang K. (2018). An obligatory anaerobic Salmonella typhimurium strain redirects M2 macrophage to the M1 phenotype. Oncol. Lett..

[28] Brundu S.F.A. (2015). Polarization and Repolarization of Macrophages. J. Clin. Cell. Immunol..

[29] Arsenault R.J., Kogut M.H. (2015). Immunometabolism and the Kinome Peptide Array: A New Perspective and Tool for the Study of Gut Health. Front. Vet. Sci..

[30] Li Y., Arsenault R.J., Trost B., Slind J., Griebel P.J., Napper S., Kusalik A.J. (2012). A Systematic Approach for Analysis of Peptide Array Kinome Data. Sci. Signal..

[31] Arsenault R.J., Kogut M.H. (2012). Chicken-Specific Peptide Arrays for Kinome Analysis: Flight for the Flightless. Curr. Top. Biotechnol..

[32] Jalal S., Arsenault R., Potter A., Babiuk L.A., Griebel P.J., Napper S. (2009). Genome to Kinome: Species-Specific Peptide Arrays for Kinome Analysis. Sci. Signal..

[33] Parikh K., Peppelenbosch M.P., Ritsema T. (2009). Kinome Profiling Using Peptide Arrays in Eukaryotic Cells. Advanced Structural Safety Studies.

[34] Ardito F., Giuliani M., Perrone D., Troiano G., Muzio L.L. (2017). The crucial role of protein phosphorylation in cell signaling and its use as targeted therapy (Review). Int. J. Mol. Med..

[35] Borsoi A., Santos L.R.D., Rodrigues L.B., Moraes H.L.D.S., Salle C.T.P., Nascimento V.P.D. (2011). Behavior of Salmonella Heidelberg and Salmonella Enteritidis Strains Following Broiler Chick Inoculation: Evaluation of Cecal Morphometry, Liver and Cecum Bacterial Counts And Fecal Excretion Patterns. Braz. J. Microbiol..

[36] Kogut M.H., Genovese K.J., He H., Arsenault R.J. (2016). AMPK and mTOR: Sensors and regulators of immunometabolic changes during Salmonella infection in the chicken. Poult. Sci..

[37] He H., Genovese K.J., Swaggerty C., Nisbet D.J., Kogut M.H. (2012). A Comparative Study on Invasion, Survival, Modulation of Oxidative Burst, and Nitric Oxide Responses of Macrophages (HD11), and Systemic Infection in Chickens by Prevalent Poultry Salmonella Serovars. Foodborne Pathog. Dis..

[38] Wu J., Pugh R., Laughlin R.C., Andrews-Polymenis H., McClelland M., Bäumler A.J., Adams L.G. (2014). High-throughput Assay to Phenotype Salmonella enterica Typhimurium Association, Invasion, and Replication in Macrophages. J. Vis. Exp..

[39] Arsenault R., Lee J.T., Latham R., Carter B., Kogut M.H. (2017). Changes in immune and metabolic gut response in broilers fed β-mannanase in β-mannan-containing diets. Poult. Sci..

[40] Trost B., Kindrachuk J., Maattanen P., Napper S., Kusalik A. (2013). PIIKA 2: An Expanded, Web-Based Platform for Analysis of Kinome Microarray Data. PLoS ONE.

[41] Szklarczyk D., Morris J.H., Cook H.V., Kuhn M., Wyder S., Simonovic M., Santos A., Doncheva N.T., Roth A., Bork P. (2016). The STRING database in 2017: Quality-controlled protein-protein association networks, made broadly accessible. Nucleic Acids Res..

[42] Kanehisa M., Sato Y., Kawashima M., Furumichi M., Tanabe M. (2015). KEGG as a reference resource for gene and protein annotation. Nucleic Acids Res..

[43] Boutet E., Lieberherr D., Tognolli M., Schneider M., Bansal P., Bridge A., Poux S., Bougueleret L., Xenarios I. (2016). UniProtKB/Swiss-Prot, the Manually Annotated Section of the UniProt KnowledgeBase: How to Use the Entry View. Adv. Struct. Saf. Stud..

[44] Zaru R., Magrane M., O’Donovan C. (2017). The UniProt Consortium From the research laboratory to the database: The Caenorhabditis elegans kinome in UniProtKB. Biochem. J..

[45] Pundir S., Martin M.J., O’Donovan C. (2017). Chapter 2. Protein Knowledgebase. Breast Cancer.

[46] Hornbeck P., Zhang B., Murray B., Kornhauser J.M., Latham V., Skrzypek E. (2014). PhosphoSitePlus, 2014: Mutations, PTMs and recalibrations. Nucleic Acids Res..

[47] Mookerjee S.A., Brand M.D. (2015). Measurement and Analysis of Extracellular Acid Production to Determine Glycolytic Rate. J. Vis. Exp..

[48] Seahorse XFp Analyzer/Agilent. https://www.agilent.com/en/products/cell-analysis/seahorse-analyzers/seahorse-xfp-analyzer.

[49] Cascales E. (2017). Inside the Chamber of Secrets of the Type III Secretion System. Cell.

[50] Jennings E., Thurston T.L., Holden D.W. (2017). Salmonella SPI-2 Type III Secretion System Effectors: Molecular Mechanisms and Physiological Consequences. Cell Host Microbe.

[51] Crhanova M., Hradecka H., Faldynova M., Matulova M., Havlickova H., Sisak F., Rychlik I. (2011). Immune Response of Chicken Gut to Natural Colonization by Gut Microflora and to Salmonella enterica Serovar Enteritidis Infection. Infect. Immun..

[52] Patel J.C., Galán J.E. (2006). Differential activation and function of Rho GTPases during Salmonella–host cell interactions. J. Cell Boil..

[53] Quilliam L.A., Lambert Q.T., Mickelson-Young L.A., Westwick J.K., Sparks A.B., Kay B.K., Jenkins N.A., Gilbert D.J., Copeland N.G., Der C.J. (1996). Isolation of a NCK-associated Kinase, PRK2, an SH3-binding Protein and Potential Effector of Rho Protein Signaling. J. Boil. Chem..

[54] Matsuzawa T., Kuwae A., Yoshida S., Sasakawa C., Abe A. (2004). Enteropathogenic Escherichia coli activates the RhoA signaling pathway via the stimulation of GEF-H1. EMBO J..

[55] Feng J., Ito M., Ichikawa K., Isaka N., Nishikawa M., Hartshorne D.J., Nakano T. (1999). Inhibitory Phosphorylation Site for Rho-associated Kinase on Smooth Muscle Myosin Phosphatase. J. Boil. Chem..

[56] Anderson C., Kendall M.M. (2017). Salmonella enterica Serovar Typhimurium Strategies for Host Adaptation. Front. Microbiol..

[57] Lopez C.A., Winter S.E., Rivera-Chávez F., Xavier M.N., Poon V., Nuccio S.-P., Tsolis R.M., Bäumler A.J. (2012). Phage-Mediated Acquisition of a Type III Secreted Effector Protein Boosts Growth of Salmonella by Nitrate Respiration. mBio.

[58] Wigley P. (2014). Salmonella enterica in the Chicken: How it has Helped Our Understanding of Immunology in a Non-Biomedical Model Species. Front. Immunol..

[59] Bratburd J.R., Keller C., Vivas E., Gemperline E., Li L., Rey F.E., Currie C.R., Hsiao A., Whiteson K. (2018). Gut Microbial and Metabolic Responses to Salmonella enterica Serovar Typhimurium and Candida albicans. mBio.

[60] Hardie D.G. (2011). AMP-activated protein kinase—An energy sensor that regulates all aspects of cell function. Genes Dev..

[61] Berg J.M., Tymoczko J.L., Stryer L. (2002). 20.3 the Pentose Phosphate Pathway Generates NADPH and Synthesizes Five-Carbon Sugars. Biochemistry.

[62] Poulsen B.R., Nøhr J., Douthwaite S., Hansen L.V., Iversen J.J.L., Visser J., Ruijter G.J.G. (2005). Increased NADPH concentration obtained by metabolic engineering of the pentose phosphate pathway in Aspergillus niger. FEBS J..

[63] Stunault M.I., Bories G., Guinamard R.R., Ivanov S. Metabolism Plays a Key Role during Macrophage Activation. https://www.hindawi.com/journals/mi/2018/2426138/.

[64] Müller A.J., Hoffmann C., Galle M., Broeke A.V.D., Heikenwalder M., Falter L., Misselwitz B., Kremer M., Beyaert R., Hardt W.-D. (2009). The *S.* Typhimurium Effector SopE Induces Caspase-1 Activation in Stromal Cells to Initiate Gut Inflammation. Cell Host Microbe.

[65] Brint E.K., Fitzgerald K.A., Smith P., Coyle A.J., Gutierrez-Ramos J.-C., Fallon P.G., O’Neill L.A.J. (2002). Characterization of Signaling Pathways Activated by the Interleukin 1 (IL-1) Receptor Homologue T1/ST2. J. Boil. Chem..

[66] Dolniak B., Katsoulidis E., Carayol N., Altman J.K., Redig A.J., Tallman M.S., Ueda T., Watanabe-Fukunaga R., Fukunaga R., Platanias L.C. (2008). Regulation of Arsenic Trioxide-induced Cellular Responses by Mnk1 and Mnk2. J. Boil. Chem..

[67] Fan C., Yang J., Engelhardt J.F. (2002). Temporal pattern of NF? B activation influences apoptotic cell fate in a stimuli-dependent fashion. J. Cell Sci..

[68] Hahn-Windgassen A., Nogueira V., Chen C.-C., Skeen J.E., Sonenberg N., Hay N. (2005). Akt Activates the Mammalian Target of Rapamycin by Regulating Cellular ATP Level and AMPK Activity. J. Boil. Chem..

[69] Buerger C., Shirsath N., Lang V., Berard A., Diehl S., Kaufmann R., Boehncke W.-H., Wolf P. (2017). Inflammation dependent mTORC1 signaling interferes with the switch from keratinocyte proliferation to differentiation. PLoS ONE.

[70] Saxton R.A., Sabatini D.M. (2017). mTOR Signaling in Growth, Metabolism, and Disease. Cell.

[71] Amano M., Nakayama M., Kaibuchi K. (2010). Rho-kinase/ROCK: A key regulator of the cytoskeleton and cell polarity. Cytoskeleton.

[72] Prakash S.K., Paylor R., Jenna S., Lamarche-Vane N., Armstrong D.L., Xu B., Mancini M.A., Zoghbi H.Y. (2000). Functional analysis of ARHGAP6, a novel GTPase-activating protein for RhoA. Hum. Mol. Genet..

[73] Behnsen J., Pérez-López A., Nuccio S.-P., Raffatellu M. (2015). Exploiting host immunity: The Salmonella paradigm. Trends Immunol..

[74] Schikora A., Virlogeux-Payant I., Bueso E., García A.V., Nilau T., Charrier A., Pelletier S., Menanteau P., Baccarini M., Velge P. (2011). Conservation of Salmonella Infection Mechanisms in Plants and Animals. PLoS ONE.

[75] Alvarado-Kristensson M., Melander F., Leandersson K., Rönnstrand L., Wernstedt C., Andersson T. (2004). p38-MAPK Signals Survival by Phosphorylation of Caspase-8 and Caspase-3 in Human Neutrophils. J. Exp. Med..

[76] Arpaia N., Godec J., Lau L., Sivick K.E., McLaughlin L.M., Jones M.B., Dracheva T., Peterson S.N., Monack D.M., Barton G.M. (2011). TLR Signaling Is Required for Salmonella typhimurium Virulence. Cell.

[77] Elsheimer-Matulova M., Varmuzova K., Sisak F., Havlickova H., Babak V., Stejskal K., Zdráhal Z., Rychlik I. (2013). Chicken innate immune response to oral infection with Salmonella enterica serovar Enteritidis. Vet. Res..

[78] Venkitanarayanan K., Thakur S., Ricke S.C. (2019). Food Safety in Poultry Meat Production.

[79] Vazquez-Torres A., Fang F.C. (2001). Salmonella evasion of the NADPH phagocyte oxidase. Microbes Infect..

[80] Choi Y.J., Park Y.J., Jeong H.O., Kim D.H., Ha Y.M., Kim J.M., Song Y.M., Heo H.-S., Yu B.P. (2012). Inhibitory Effect of mTOR Activator MHY1485 on Autophagy: Suppression of Lysosomal Fusion. PLoS ONE.

